# ATM kinase sustains breast cancer stem-like cells by promoting ATG4C expression and autophagy

**DOI:** 10.18632/oncotarget.15537

**Published:** 2017-02-20

**Authors:** Martina Antonelli, Flavie Strappazzon, Ivan Arisi, Rossella Brandi, Mara D’Onofrio, Manolo Sambucci, Gwenola Manic, Ilio Vitale, Daniela Barilà, Venturina Stagni

**Affiliations:** ^1^ Instituto di Ricovero e Cura a Carattere Scientifico (IRCCS) Fondazione Santa Lucia, Rome, Italy; ^2^ Department of Biology, University of Rome ‘Tor Vergata’, Rome, Italy; ^3^ Genomics Facility, European Brain Research Institute (EBRI) ‘Rita Levi-Montalcini’, Rome, Italy; ^4^ Neuroimmunology Unit, Fondazione Santa Lucia, IRCCS, Rome, Italy; ^5^ Regina Elena National Cancer Center Institute, Rome, Italy

**Keywords:** ATM kinase, breast cancer stem cells (BCSCs), mammospheres, autophagy, ATG4

## Abstract

The efficacy of Ataxia-Telangiectasia Mutated (ATM) kinase signalling inhibition in cancer therapy is tempered by the identification of new emerging functions of ATM, which suggests that the role of this protein in cancer progression is complex. We recently demonstrated that this tumor suppressor gene could act as tumor promoting factor in HER2 (Human Epidermal Growth Factor Receptor 2) positive breast cancer. Herein we put in evidence that ATM expression sustains the proportion of cells with a stem-like phenotype, measured as the capability to form mammospheres, independently of HER2 expression levels. Transcriptomic analyses revealed that, in mammospheres, ATM modulates the expression of cell cycle-, DNA repair- and autophagy-related genes. Among these, the silencing of the autophagic gene, autophagy related 4C cysteine peptidase (ATG4C), impairs mammosphere formation similarly to ATM depletion. Conversely, ATG4C ectopic expression in cells silenced for ATM expression, rescues mammospheres growth. Finally, tumor array analyses, performed using public data, identify a significant correlation between ATM and ATG4C expression levels in all human breast cancer subtypes, except for the basal-like one.

Overall, we uncover a new connection between ATM kinase and autophagy regulation in breast cancer. We demonstrate that, in breast cancer cells, ATM and ATG4C are essential drivers of mammosphere formation, suggesting that their targeting may improve current approaches to eradicate breast cancer cells with a stem-like phenotype.

## INTRODUCTION

The cancer stem cell hypothesis proposes that cancers arise from and are maintained by a small population of cancer-initiating cells residing within tumor mass. These cells are characterized by the re-expression of stem cell markers and self-renewal potential, and have therefore been named cancer stem cells (CSCs) or more exactly cancer stem-like cells. [1]. In recent years, CSCs have been identified in multiple cancers, including breast cancer, and they were shown to be particularly resistant to conventional anticancer therapy, which may contribute to treatment failure and tumor relapse [2]. The fate of cancer stem cells is determined by the “stem cell niche” in the tumor, which comprises stromal cells, cytokines, and growth factors. Moreover this niche is characterized by starvation and hypoxic conditions that are considered critical niche factors to promote invasive growth of tumors. The observation that CSCs have a specific regulation by the microenvironment suggests the presence of a peculiar metabolic demand and a specific response to environmental stresses in these cells compared to the bulk tumor [3]. Molecular mechanisms involved in their regulation, maintenance and resistance to therapies are peculiar and specific for this subpopulation of cells in the tumor and so targeting strategies for the destruction of CSCs specific signalling pathways provides a novel opportunity for cancer research [3].

Ataxia-Telangiectasia Mutated (ATM) is a multifunctional kinase that plays complex and controversial roles in cancer. ATM is historically considered a tumor suppressor gene for its central role in the DNA damage response (DDR) [4, 5]. Indeed, this kinase is considered as a good target for cancer therapies and, consistently with its DDR function, also drives the therapeutic resistance of CSCs [6–8]. We have recently identified ATM as a promoter of HER2 tumorigenicity in breast cancer [9], suggesting a dual function of ATM in cancer. In particular, we showed that abrogating ATM function significantly impaired HER2-dependent tumorigenicity *in vitro* and *in vivo* also uncovering a novel cancer-related function of ATM as regulator of HER2 receptor stability [9]. Importantly, ATM plays non-nuclear functions in addition to the DDR signalling that may contribute to its dual, opposing role in cancer [6–8]. The emerging role of ATM in the regulation of autophagy is intriguing [10–12], although its impact on cancer progression has been poorly investigated so far. Interestingly, by using *Atm*^−/−^*Becn1*^+/−^ mice model, it was demonstrated that the genetic inactivation of the autophagic gene Beclin 1 results in a significant delay of lymphoma and leukaemia onset normally occurring in *Atm*-deficient mice, due to the rescue of mitochondrial abnormalities and not of the DDR [13]. This observation supports the hypothesis that ATM kinase and autophagy could talk each other in the regulation of cancer progression.

The role of autophagy in carcinogenesis remains elusive. Autophagy may promote or counteract tumor initiation and progression depending on the specific context [14]. Recent evidence support the idea that autophagy could act as a cytoprotective process to augment CSC survival under conditions of nutrient or growth factor starvation, metabolic stress, and hypoxia within the “stem cell niche” in the tumor [15, 16]. Consistently, autophagy is an important driver of stem-like phenotype in breast cancer [17–19] and autophagic genes such as ATG4A and Beclin 1 play a role in breast (B)CSC maintenance, further supporting the idea that BCSCs employ autophagy for promoting their survival and growth [18, 19].

Overall these evidences led us to investigate the role of ATM kinase in the regulation of the stem-like phenotype in breast cancer. Herein, we utilised the preparation of 3D spheroid cultures, also known as “mammospheres” [20], as functional assay to enrich for a population of cells with a stem-like phenotype to investigate the role of ATM in the regulation of Breast Cancer Stem-like cells. Our results give novel insights into the molecular mechanisms underlying mammosphere formation attributing a previously unrecognised role in this process to the crosstalk between ATM kinase and ATG4C autophagic gene in breast cancer progression.

## RESULTS

### ATM expression determines the ability to form mammospheres and promotes breast cancer-stem like phenotype

**Figure 1:**
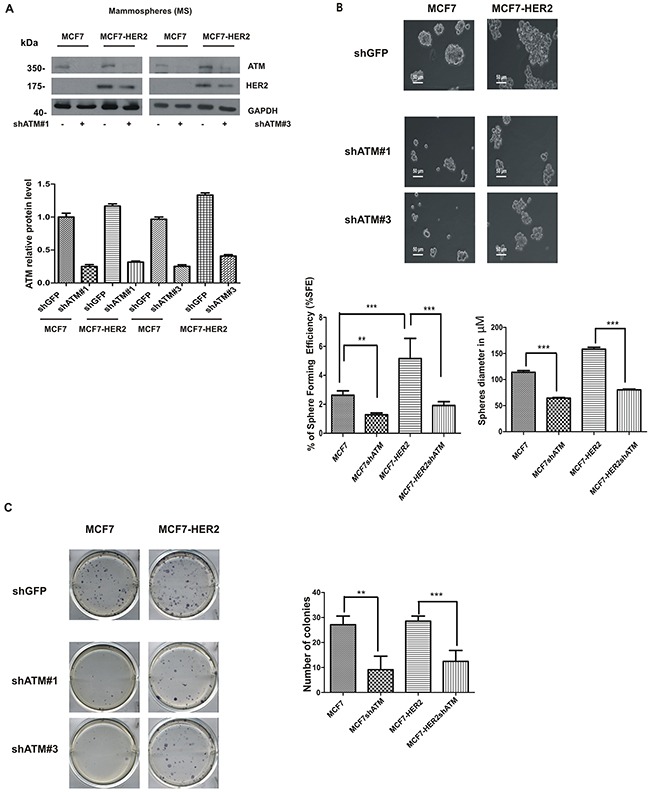
ATM expression promotes mammospheres formation MCF7 and MCF7-HER2 breast cancer cell lines were infected with lentivirusexpressing two different RNA interference for ATM (shATM #1 and shATM #3) or a control sequence (shGFP). **A**. Representative Western Blot analysis of ATM and HER2 protein levels in mammospheres (MS); GAPDH was used as loading control (upper panel). The graph represent quantification of ATM protein levels and it was determined using the ImageJ software. Results are indicated as mean±s.d. for three independent experiments **B**. Single cells were plated in ultralow attachment plates as described in Materials and methods section, so that cells with stem cell properties were allowed to grow as non-adherent spheroids (mammospheres). Images of the mammospheres were captured on day 7. Representative phase-contrast images of mammospheres are shown. Bars denote 50 μm. Numbers of the mammospheres (diameter>50 μm) were counted, and the % of Sphere Forming Efficiency (%SFE) was calculated based on the numbers of cells that were initially seeded as mean±s.d. for three independent experiments performed with both targeting sequences for ATM (shATM#1 and shATM#3). The diameter of mammosphere (in μm) was quantified using I.A.S software (Delta Sistemi, Italy). **C**. Mammospheres were dissociated by trypsin digestion and 600 cells/well were seeded in 6-well plates in differentiating media (mammospheres medium + 5% FBS serum). Colony formation was assessed 7 days later and stained with MTT (left panel). The number of colonies are expressed as mean±s.d. for three independent experiments performed with both targeting sequences for ATM (shATM #1 and shATM #3) . Student's t-test **P<0.01, ***P<0.001) (right panel).

**Figure 2:**
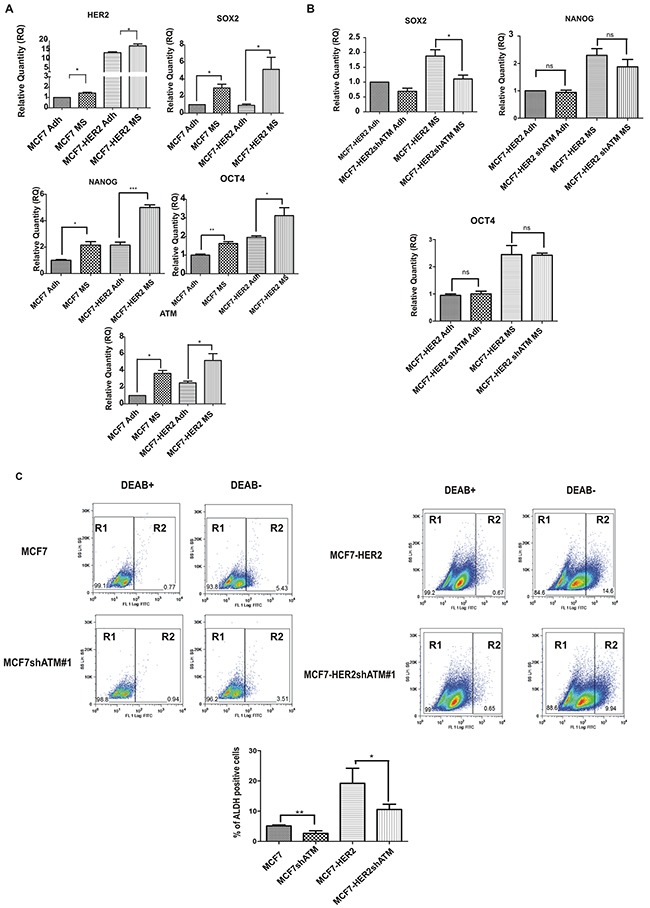
ATM expression promotes stem-like phenotype **A**. The expression of HER2, ATM and SOX2, OCT4 and NANOG mRNA levels in non-infected Mammospheres (MS) and Adherent (Adh) cells was investigated using by quantitative Real-Time PCR. Relatives quantities (RQ) were calculated to TBP (TATA-box Binding Protein) and are relative to MCF7 adherent parental cells (Adh). Results are expressed as the mean±s.d. for at least three independent experiments and analyzed using Student's t-test (*P<0.05, ***P<0.001). **B**. The expression of SOX2,OCT4 and NANOG mRNA levels was investigated as in infected Mammospheres (MS) with lentivirus expressing control interference (shGFP) or shATM. **C**. Indicated cells were assayed for ALDH activity utilizing the ALDEFLUOR™ assay (STEMCELL™ TECHNOLOGIES). Cells incubated with ALDEFLUOR substrate (BAAA) and the specific inhibitor of ALDH, diethylaminobenzaldehyde (DEAB), were used to establish the baseline fluorescence of these cells (R1) and to define the ALDEFLUOR-positive region (R2). Incubation of cells with ALDEFLUOR substrate in the absence of DEAB (DEAB-) induces a shift in BAAA fluorescence defining the ALDEFLUOR-positive population (R2). The quantification of ALDH-positive cells in each breast cancer cell line is shown (lower panel). Error bars indicate the s.d. from three independent experiments with both targeting sequences for ATM (shATM#1 and shATM#3).

Recent works suggest that HER2 is a driver of cancer stem-like phenotype in luminal estrogen receptor-positive (ERp) breast cancers, in the absence of HER2 gene amplification, and in breast cancers with amplification of HER2 receptor [21, 22]. In order to analyse the impact of ATM kinase on formation of cells with a stem-like phenotype in breast cancer, we choose as cancer model system: 1) mammospheres derived from luminal estrogen receptor-positive HER2-low breast cancer cell line (MCF7); 2) mammospheres derived from luminal estrogen receptor-positive HER2-overexpressing breast cancer cell line (MCF7-HER2). To avoid off-target effects, ATM expression was genetically downregulated by two specific shRNA interference in both cell lines, using lentiviral vectors, (Figure 1A) and *in vitro* mammospheres formation was assayed by measuring the ability to grow in low serum and anchorage independent conditions [20] (Figure 1B). In these experiments, we observed that the downregulation of ATM led to a decrease in mammospheres number and size in both MCF7 cell lines with the two ATM target sequences (Figure 1B). On average, 200 mammospheres formed from 8000 MCF7 cells (∼2,5% of Sphere Forming Efficiency, SFE) seeded under serum-free suspension conditions and 400 mammospheres formed from 8000 MCF7-HER2 cells (∼5 %, SFE) (Figure 1B), which is in accordance with the crucial role of HER2 receptor as promoter of mammospheres formation. Interestingly, the silencing of ATM caused a ∼50% reduction in mammospheres number and diameter in both cell lines (Figure 1B). In addition to sphere formation, the colony formation capacity of dissociated mammospheres seeded in 2D adherent plate was analysed. As shown in Figure 1C, silencing of ATM expression significantly reduced the number of colonies grown in both cell lines. It is well established that, cells grown as mammospheres, compared to cells grown in adherent conditions, significantly upregulate the expression of genes driving the stem like phenotype [23]. In line with this observation, we could show that the expression of HER2 and of several stem cell markers such as SRY-box 2 (SOX2), POU class 5 homeobox 1 (POU5F1; best known as OCT4) and Nanog homeobox (NANOG) was elevated in mammospheres, derived from both cell lines, compared to adherent cells (Figure 2A). Similarly, the level of ATM mRNA was upregulated in mammospheres, when compared to adherently cultured cells (Figure 2A). However, the selective silencing of ATM expression, with both target sequences using lentiviral vectors, resulted in the impairment of SOX2 , but not of OCT4 and NANOG mRNA expression in mammospheres (Figure 2B).

To further investigate the role of ATM expression in driving the stem-like phenotype in breast cancer, we evaluated, by flow cytometry, the percentage of aldehyde dehydrogenase (ALDH) activity-positive cell population in our cell lines; indeed ALDH activity is validated as a well-known marker of the sub-population of cells with stem-like characteristics [24]. Consistently with previous reports [10], HER2 overexpression enhanced ALDH activity (Figure 2C) [10]. More interestingly, we could show that the genetic downregulation of ATM expression, with both shRNA sequences, resulted in the reduction of ALDH activity in both cell lines (Figure 2C and Supplementary Table 1). Overall, while these data indicate a role of ATM expression in the modulation of mammospheres formation, probably this is independent of HER2 expression levels. They also suggest that ATM exerts a mild effect on the expression of stemness markers.

### Identification of ATM-dependent regulated gene set in mammospheres

**Figure 3:**
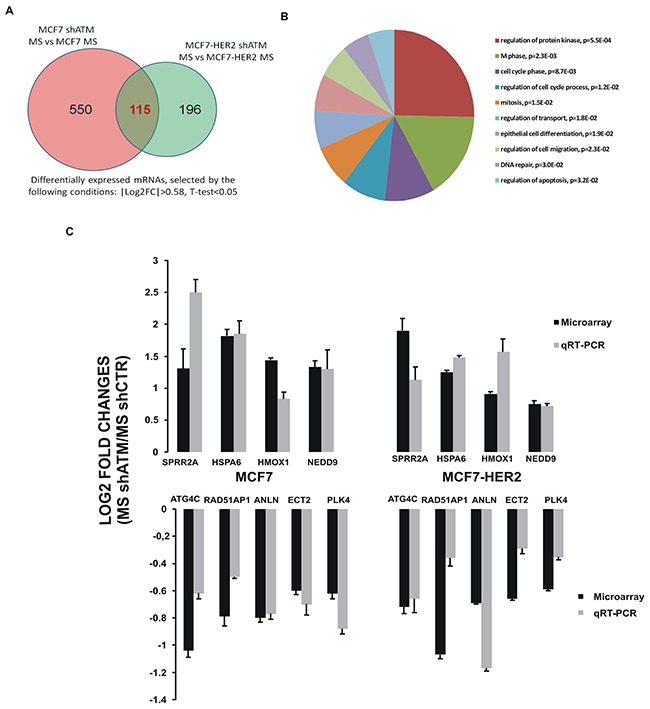
Gene expression profiling of mammospheres with or without interfering ATM expression **A**. Number of differentially expressed mRNAs between mammospheres silenced for ATM and control mammospheres, obtained from three independent experiment performed with both targeting sequences for ATM (shATM#1 and shATM#3) and selected by the following conditions: |Log2FC|>0.58, T-test<0.05. In the Figure are shown the number of differentially expressed genes in mammospheres derived from MCF7 and from MCF7-HER2 cells. The intersection area encloses 115 common genes between mammospheres derived from both cell lines. **B**. Gene Ontology analysis of intersection set (115 mRNAs), obtained by the DAVID online tool. **C**. Microarray and qRT-PCR comparison. Log2 Fold Change of 9 genes between mammospheres shATM vs shCTR detected by microarray (black lines) were compared with those measured by qRT-PCR (grey lines). Positive values represent gene expression upregulation and negative values downregulation in mammospheres silenced for ATM gene (shATM) compared to control cells (shCTR). qRT-PCR results were normalized with TBP. Error bars indicate standard deviations of at least three independently performed experiments.

To further uncover the molecular mechanism involved in ATM-mediated regulation of mammospheres formation, we performed a gene expression analysis. We used two different RNA interference sequences targeting ATM and we performed the experiment as biological triplicate: three independent lentiviral infections were carried out on both cell lines (MCF7 and MCF7-HER2) silenced or not for ATM, grown as mammospheres. Microarray based expression profiling, pooling all the experiments, revealed that 550 probes for MCF7 and 196 probes for MCF7-HER2 were significantly expressed in mammospheres derived from cells silenced for ATM in a differential fashion compared to those obtained from control cell lines, interfered with shGFP (Figure 3A). As ATM impairs mammospheres formation independently of HER2 expression (Figure 1), we focused our analysis on the differential expression of 115 genes intersected in MCF7 and MCF7-HER2, which are downregulated or upregulated upon ATM expression silencing (Figure 3A). We hypothesized that these genes could be responsible for ATM-dependent reduction of mammospheres formation irrespective of HER2 expression levels. Functional annotation of these 115 genes using DAVID (Database for Annotation, Visualization and Integrated Discovery) revealed that they are mainly associated to mitosis, regulation of protein kinase, cell cycle, DNA repair and cell death (Figure 3B). In Supplementary Table 2, we selected the first 10 upregulated and first 10 downregulated genes in mammospheres shATM versus shCTR, involved in these pathways (Supplementary Table 2). Validation of the expression pattern of 9 genes (4 upregulated and 5 downregulated), from our 20 selected genes, obtained by microarray analysis was performed by qRT-PCR (Figure 3C and Supplementary Figure 1A-1B). In all cases, the trend of fold change of expression identified in the microarrays studies was confirmed by qRT-PCR experiments (Figure 3C). Interestingly, when we compared the expression of our selected genes in mammospheres with respective adherent parental cell lines, we observed that silencing ATM in mammospheres resulted in an expression profile more similar to that of adherent parental cells, supporting the idea that ATM expression could modulate essential genes involved in mammospheres formation (Supplementary Figure 1A-1B) [19].

### Autophagic gene ATG4C promotes mammospheres formation

It was recently reported that some autophagic regulator genes, such as Beclin-1 and ATG4A, modulated the autophagic flux in mammospheres derived from MCF7 cells, and that the deregulation of this feature impaired mammospheres forming capacity [18, 19]. By analyzing our microarray and qRT-PCR expression data, we observed that the expression of the autophagic gene ATG4C was significantly downregulated in mammospheres interfered for ATM expression, compared to control ones (Supplementary Table 2 and Figure 3C). Moreover, ATG4C mRNA expression was upregulated in cells grown as mammosphere compared to the ones grown in adherent conditions (Supplementary Figure 1A). Overall, these data suggest that ATG4C may promote mammospheres formation.

**Figure 4:**
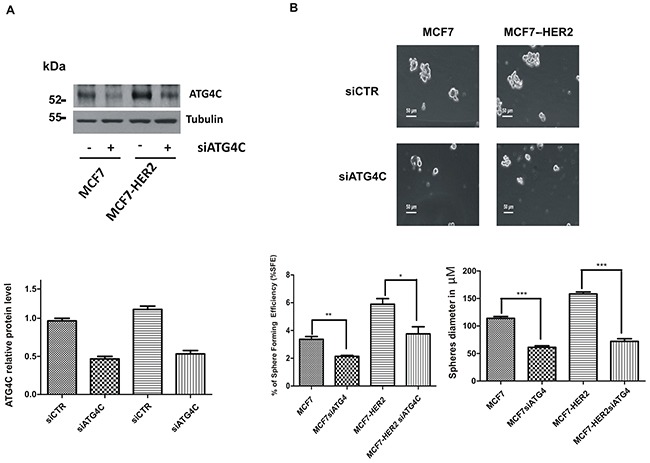
ATG4C expression promotes mammospheres formation MCF7 and MCF7-HER2 breast cancer cell lines were transfected with ATG4C siRNA or a control sequence (SignaliSilence ®,Cell Signaling) using Lipofectamine ® 3000 Reagent (Life Technologies). **A**. Representative Western Blot analysis of ATG4C protein levels in mammospheres (MS); Tubulin was used as loading control (upper panel). **B**. Single cells were plated in ultralow attachment plates as described in Materials and Methods section, so that cells with stem cell properties were allowed to grow as mammospheres. Images of the mammospheres were captured on day 7. Representative phase-contrast images of mammospheres are shown (upper). Bars denote 50 μm. Numbers of the mammospheres (diameter>50 μm) were counted, and the %SFE was calculated based on the numbers of cells that were initially seeded (Bottom). mean±s.d. for three independent experiments and analysed using Student's t-test (*P<0.05, **P<0.01, ***P<0.001). The diameter of mammosphere (in μm) was quantified using I.A.S software (Delta sistemi, Italy).

**Figure 5:**
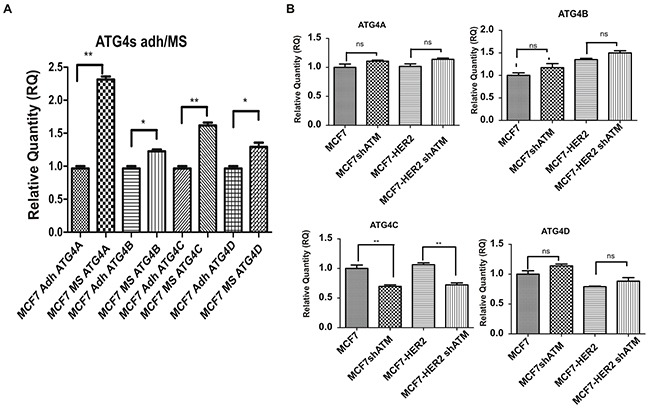
Downregulation of ATM expression selectively impinges on ATG4C expression **A**.The expression of ATG4A, B, C and D mRNA levels in non-infected Mammospheres (MS) and Adherent (Adh) cells was investigated using by quantitative Real-Time PCR. Relatives quantities (RQ) were calculated to TBP (TATA-box Binding Protein) and are relative to MCF7 adherent parental cells (Adh). Results are expressed as the mean±s.d. for at least three independent experiments and analyzed using Student's t-test (*P<0.05, **P<0.01). **B**. The expression of ATG4A, B, C and D mRNA levels was investigated as in (A) in infected Mammospheres (MS) with lentivirus expressing control interference (shGFP) or shATM.

To further validate our hypothesis we downregulated ATG4C expression by specific RNA interference selective for this isoform (Figure 4A and Supplementary Figure 2C). Remarkably, we could show that the downregulation of ATG4C expression impairs mammospheres forming ability (Figure 4B), similarly to what previously observed upon ATM expression silencing (Figure 1A-1B). ATG4C is one on the four members of ATG4s protease family (including ATG4A, B, C and D); the family members share similar structure but have very well established different functions [25]. Recently, ATG4A has been identified as a modulator of mammospheres formation [18]. Accordingly, we could show that the expression of all ATG4 family members is enhanced, although to different extent, in mammospheres compared to adherent cells. Importantly ATG4A was the ATG4 family members most strongly up regulated in our models (Figure 5A). More interestingly, ATM does not affect the mRNA levels of ATG4A, B and D neither in MCF7 nor in MCF7-HER2 cells (Figure 5B) driving the conclusion that ATM selectively impinges on ATG4C expression.

### ATM modulates ATG4C levels and sustains autophagic flux in mammospheres

**Figure 6:**
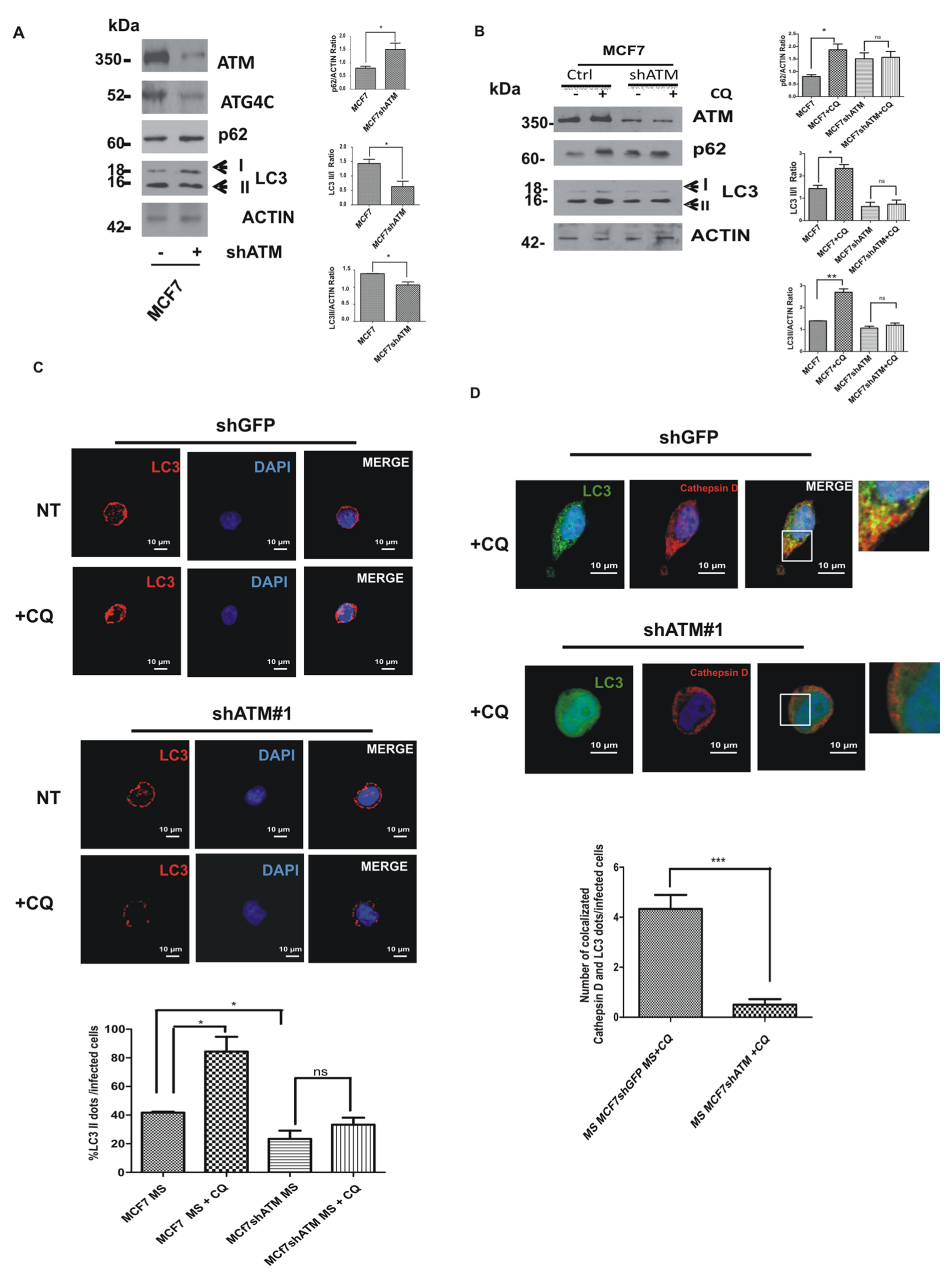
ATM expression regulates ATG4C levels and autophagic flux in mammospheres (MS) **A**. Representative Western Blot analysis of ATM, ATG4C, p62 and LC3 protein levels in mammospheres (MS) derived from MCF7 cell lines, with or without shATM. Actin was used as loading control. The graphs represented quantification of LC3 II/I ratio, LC3II/actin and p62/actin ratio was determined using the ImageJ software. Results are indicated as mean±s.d. for three independent experiments performed with both targeting sequences for ATM (shATM#1 and shATM#3) and analysed using Student's t-test (*P<0.05, **P<0.01, ***P<0.001). **B**. Representative Western Blot analysis of p62 and LC3 proteins in mammospheres (MS) with or without shATM and treated with CQ (20 μM, 30 min). Actin was used as loading control. The graphs represented quantification of LC3 II/I ratio, LC3II/actin and p62/actin ratio was determined using the ImageJ software. Results are indicated as mean±s.d. for three independent experiments performed with both targeting sequences for ATM (shATM#1 and shATM#3) and analysed using Student's t-test (*P<0.05). **C**. Representative image, using confocal microscopy, ofthe formation of autophagosome assayed by immunofluorescence for endogenous LC3 protein in MCF7 cells seeded from dissociated mammospheres. Mammospheres were treated or not with choloroquine CQ (20 μM, 30 min) for analysing autophagic flux. The graph show the accumulation of LC3 dots per infected cells. Results are expressed as the mean±s.d. for at least three independent experiments performed with both targeting sequences for ATM (shATM#1 and shATM#3) and analysed using Student's t-test (*P<0.05). **D**. Representative image, using confocal microscopy, of the lysosomal degradation of autophagosomes assayed by immunofluorescence for endogenous LC3 and Cathepsin D proteins in MCF7 cells seeded from dissociated mammospheres. Mammospheres were treated with choloroquine CQ (20 μM, 30 min) in order to visualize the autophagosome degradation (co-localization LC3-II dots with Cathepsin D). The graph indicates a clear reduction of LC3-II dots co-localizing with Cathepsin D in ShATM infected cells. Results are expressed as the mean±S.D of at least three independent experiments performed with both targeting sequences for ATM (shATM#1 and shATM#3) and analysed using Student's t-test ( ***P<0.001).

ATG4C is a member of ATG4s proteases that are responsible for the cleavage of Microtubule-associated protein 1A/1B-light chain 3 (LC3-I) into LC3-II protein, which is then lipidated and recruited to autophagosomal membranes [25]. Tracking the conversion of LC3-I to LC3-II is indicative of ATG4s activity and thus of autophagic activity [26]. We therefore investigated whether the loss of ATM-dependent regulation of ATG4C expression level may result in a defect in autophagosome formation in mammospheres upon ATM silencing. To quantify autophagosome formation we analysed the expression and the processing of LC3 protein, a well-known marker of autophagy [27]. Western blot analysis showed that the downregulation of ATM expression in mammospheres triggers the reduction of ATG4C protein levels (Figure 6A), and the impairment of LC3-I conversion into LC3-II monitored through quantification of LC3*-*II*/* LC3*-*I ratio (Figure 6A).

To further assess the effect of ATM-dependent regulation of ATG4C on autophagic response we checked the levels of the autophagic receptor p62/SQSTM1, an indicator of the autophagic flux which is known to be degraded following autophagy induction [28, 29]. Interestingly the downregulation of ATM expression in mammospheres, resulted in the accumulation of p62 compared to control cells (Figure 6A). Since autophagosome formation can result either from increased *de novo* autophagosome biosynthesis or from the inhibition of the autophagic flux, we distinguished between these two possibilities using the lysosomal inhibitor chloroquine (CQ). Interestingly, in control condition, as expected, the CQ treatment blocks autophagic flux after LC3-II formation and before p62 degradation, resulting in an increased in LC3-II and p62 levels, (Figure 6B). Conversely, CQ treatment failed to induce an increase in LC3-II and p62 levels in ATM silenced mammospheres (Figure 6B) suggesting a block in the autophagic flux in shATM cells.

We confirmed these results by performing a confocal microscopy analysis of LC3 protein in mammospheres downregulated or not for ATM. In normal condition LC3 protein is cytosolic whereas it appears as “puncta” when autophagy is induced (LC3-II form). We could show a marked increase of LC3 dots in mammospheres compared to adherent cells in basal condition (Supplementary Figure 2A). Interestingly, consistently with western blot analysis (Figure 6A-6B), silencing of ATM expression drives a dramatic reduction in the formation of LC3-II dots in mammospheres (Figure 6C). To further investigate whether the reduction in the number of LC3 dots may be due to differences in the autophagic flux, MCF7 cells and derived mammospheres, were treated with Chloroquine (CQ). Efficacy of this treatment was confirmed by an increase in LC3-II dots number/cells in response to CQ in control adherent cells and control mammospheres (Figure 6C and Supplementary Figure 2A). Conversely, shATM did not increase LC3 dots formation after chloroquine treatment in mammospheres, indicating that the downregulation of ATM expression induces a decrease in the autophagic flux in this context (Figure 6C), which confirmed data obtained with western blot analysis.

We next decided to check for autophagosome degradation. To this end, we quantified LC3-II dots co-localized with the lysosomal protease Cathepsin D, following CQ treatment. As expected, we found a significant reduction of LC3-II dots fused with lysosomes in shATM cells compare to control cells (Figure 6D). Of note, ATG4A, ATG7 and ATG5-12 are stable following shATM or siATG4C treatments (Supplementary Figure 2B-2C). These data indicate a specific and unique effect of ATM on ATG4C protein. Overall these results suggest that ATM expression sustains ATG4C levels and thus the autophagic response in mammosphere context.

### Restoration of ATG4C expression rescues ATM ability to form mammospheres

**Figure 7:**
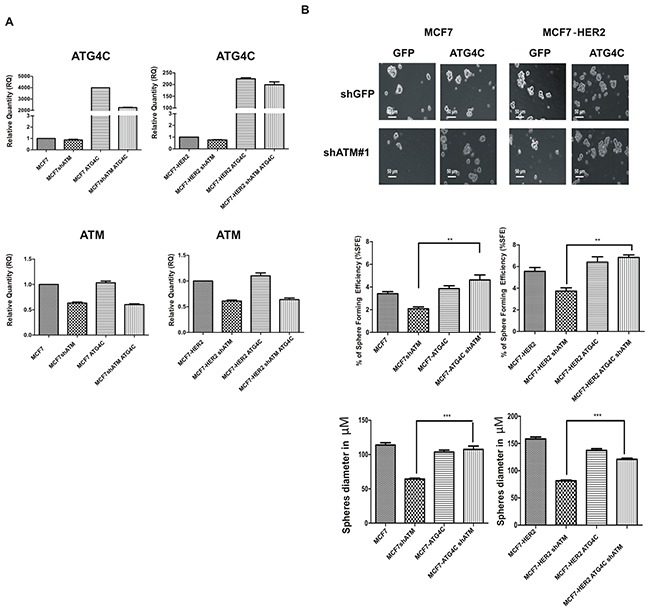
ATG4C expression rescues mammospheres formation ability in ATM interfered cells MCF7 and MCF7-HER2 breast cancer cell lines transfected with construct overexpressing human pCMV3-hATG4C-HA (ATG4C) and control GFP overexpressing construct, using Lipofectamine ® 3000 Reagent (Life Technologies) **A**. The expression of ATG4C mRNA levels in Mammospheres (MS), was investigated by quantitative Real-time PCR. Relatives quantities (RQ) were calculated to TBP and are relative to control MCF7 mammospheres. Results are expressed as the mean±s.d. for at least three independent experiments. **B**. Single cells were plated in ultralow attachment plates as described in Materials and methods section, so that cells with stem cell properties were allowed to grow as non-adherent spheroids (mammospheres). Images of the mammospheres were captured on day 7. Representative phase-contrast images of mammospheres are shown (upper). Bars denote 50 μM. Numbers of the mammospheres (diameter>50 μM) were counted, and the %SFE was calculated based on the numbers of cells that were initially seeded (Bottom). Mean±s.d. for three independent experiments performed with both targeting sequences for ATM (shATM#1 and shATM#3) and analysed using Student's t-test ( **P<0.01, ***P<0.001). The diameter of mammosphere (in μM) was quantified using I.A.S software (Delta sistemi, Italy).

To further validate the functional link between ATM and ATG4C expression in the modulation of autophagy and mammospheres formation, we overexpressed ATG4C in mammospheres previously silenced for ATM expression. In Figure 7A we confirmed by RT-PCR the overexpression of ATG4C in MCF7 and MCF7-HER2 cell lines interfered with shCTR and shATM constructs (Figure 7A). Importantly, the overexpression of ATG4C in cells silenced for ATM expression, significantly rescued the percentage of spheres formation (%SFE) of shATM cells which turn to be comparable to the one of shCTR cells (Figure 7B). This result demonstrated that there is a strong correlation between ATM-dependent regulation of ATG4C expression level and ATM-dependent regulation of mammospheres formation. This connection was independent of HER2 receptor expression levels as it can be observed in both cell lines. We cannot exclude that other proteins could be directly or indirectly involved in this regulation. Interestingly, re-expression of ATMwt in shATM mammospheres rescues ATG4C levels and mammosphere formation (Supplementary Figure 3A and 3B) further confirming the correlation between mammosphere formation and expression levels of ATM and ATG4C mRNA.

### ATM and ATG4C gene expression correlation in breast cancer human samples

To further evaluate the clinical significance of our findings, we asked whether we could identify a significant correlation between ATM and ATG4C expression in breast cancer human samples. To this aim we took advantage of The Cancer Genome Atlas (TCGA) microarray data on of breast cancer samples. To quantify the correlation, we downloaded normalized genes microarray data from the TCGA Data Coordination Center [30] and computed the Pearson correlation coefficients and corresponding *P* values (Table 1). Highlighting ATM expression showed a significant positive correlation with the expression of autophagic gene ATG4C among microarray data derived from 511 breast cancer samples (Table 1). This observation suggests that the functional correlation between ATM and ATG4C genes in mammospheres could have also a significant relevance in breast cancer patients.

**Table 1:** Correlation of ATM with ATG4C gene in BRCA samples from TCGA cancer atlas data

TCGA sample group (n)	Pearson correlation	P-value	Bonferroni corrected P-value
All BRCA (511)	0.26	**2.70E-09**	**1.35E-08**
Basal (95)	0.15	1.26E-01	6.30E-01
HER2 (58)	0.37	**3.55E-03**	**1.78E-02**
Luminal A (231)	0.17	**9.60E-03**	**4.80E-02**
Luminal B (127)	0.3	**3.29E-04**	**1.65E-03**

We computed the Pearson correlation coefficients and corresponding uncorrected and Bonferroni corrected P-values between ATM and ATG4C genes in expression data from 511 breast cancer samples extracted from the TCGA Cancer Atlas. Statistically significant results shown in bold font (P<0.05).

Breast cancer is a very heterogeneous disease. To further explore the relationship between ATM and ATG4C genes we calculated the correlations between ATM and ATG4 genes in all four subtypes of breast cancer based on published classifications [30]: two estrogen receptor (ER)-positive subtypes separated mainly by relatively low (luminal A) and high (luminal B) expression of proliferation-related genes, a subtype enriched for HER2-amplified tumors [human epidermal growth factor receptor 2 (HER2)-enriched], a subtype associated with triple-negative [lacking expression of ER, progesterone receptor (PR), and HER2] tumors (basal-like). Positive correlation between ATM and ATG4C expression was statistically significant in all subtypes, except for the basal like subtype, suggesting that the functional link between ATM and ATG4C genes could have a clinical significance (Table 1).

## DISCUSSION

Although our knowledge concerning new functions of ATM kinase has greatly increased during the last decade, their exact role in carcinogenesis and cancer therapies remains elusive. We previously demonstrated that ATM promotes HER2-dependent tumorigenesis in breast cancer, reporting also a context-dependent role of ATM targeting in cancer [9]. In this study, we described for the first time a mechanism through which ATM regulates autophagy in a fashion independent on HER2, which has crucial implications in breast cancer progression. In particular, we showed that: (1) ATM expression is induced in mammospheres culture as compared to parental adherent breast cancer cell lines, and (2) ATM depletion with a small interfering RNA leads to impaired mammospheres formation. Interestingly, we put in evidence that ATM expression sustains SOX2 expression and ALDH activity without significantly impinging on other stemness markers such as NANOG and OCT4 (Figure 2) suggesting the existence of mechanisms of modulation of mammosphere formation by ATM other than the regulation of the stemness genes. To clarify this issue, we performed microarray experiments revealing that, in mammospheres, ATM regulates the expression of genes involved in the control of DNA repair and cell division, which is consistent with the central role of ATM as guardian of the genome [4]. Moreover, in this experiment we showed that other ATM-dependent function could be involved in the regulation of Breast cancer stem-like phenotype. In particular, we focused our attention on the autophagic gene ATG4C. Indeed, autophagy is a well-established pro-survival mechanism for BCSCs maintenance [17][18, 19] suggesting the hypothesis that ATM may modulate BCSCs via ATG4C. Accordingly, we observed by western blot and real time PCR that the depletion of ATM leads to the decrease in mRNA and protein levels of ATG4C. The ATM-dependent downregulation of ATG4C expression correlates very well with an impairment of the autophagic flux in mammospheres silenced for ATM. Consistently, we showed that silencing of ATG4C impairs mammospheres formation similarly to the silencing of ATM. More interestingly, ATG4C overexpression rescues mammospheres defects induced by ATM down regulation. These findings provide the first evidence on a link between the expression of ATM and the autophagic gene ATG4C. In previous studies, ATM and ATG4C were reported to have a tumor-suppressive role [31, 32]. Here, we demostrated that these proteins promote stem-like phenotype in breast cancer, suggesting that ATM kinase and autophagy could play also a tumorigenic role in breast cancer.

In addition, in this study, we demonstrated that ATM specifically regulates ATG4C isoform in mammospheres, without affecting other members of ATG4 family, even though the precise molecular mechanism deserves further investigation. Little information is reported about the transcriptional regulation of the ATG4 proteases. Some observations indicated that ATG4C could be transcriptionally regulated by p53 upon DNA damage activation, and that ATG4C mRNA is modulated by miR-376b upon starvation and rapamycin-induced autophagy [33, 34]. Future experiment will be launched to clarify whether ATM regulates ATG4C expression through p53 or via the modulation of miR-376b.

Moreover, we cannot rule out that ATM may regulate also ATG4 protein levels and activity. Interestingly, ATG4s proteins are reported to be regulated by ROS during starvation [35]. According to this study, the Cys81 residue near the catalytic site of ATG4 is a direct oxidation target by H2O2 and the oxidation of this residue inhibits ATG4 protease activity. This, in turn, prevents the delipidation of LC3 without affecting the C-Terminal processing of LC3 by ATG4, thus leading to increased autophagosome formation [35]. It was proposed that cytosolic ATM regulates autophagy via its activation upon ROS rather than DNA damage induction [36–39]. This evidence prompts us to speculate that the selective ATM-dependent regulation of ATG4C and autophagy in mammospheres may be ascribed to differences in ROS regulation between cells grown in adherent conditions or as mammospheres. Interestingly, ATM dependent regulation of ROS plays a critical role in hematopoietic stem cell (HSC) maintenance [40]. In this study, the treatment of *Atm*^−/−^ mice with antioxidant N-acetylcystine (NAC) restored the HSC pool, confirming the critical role of ROS regulation by ATM for stemness [40]. So, we could speculate that ATM could act as a ROS sensor modulating the autophagic flux according to ROS levels in different populations of cells within the tumor. Further experiments are required to clarify whether ATM activity is involved in the regulation of the stem-like phenotype, and whether ROS- and/or DNA damage-mediated ATM activation are essential for the regulation of this phenotype. Finally, we showed that ATG4A, ATG7 and ATG5-ATG12 protein levels are unaffected by shATM treatment (Supplementary Figure 2B), supporting the idea that defects in autophagic flux mainly rely on ATG4C, even though the elucidation of the molecular mechanism requires further experimental investigations.

Interestingly, the findings reported in this study suggest that ATM may represent a novel candidate target to impair the autophagic activity in Breast Cancer Stem-like cells independently of HER2. Indeed, our results suggest that ATM targeting severely impinges not only on the DDR, as previously reported [41–43], but also on autophagy functionality, which is required for the homeostasis of the specific subset of breast cancer cells. Along similar lines, we surmise that ATG4C could also represent a valuable molecular target, as demonstrated by a large set of evidences suggesting that anti-autophagy compounds are effective in suppressing tumor growth and countering tumor resistance to chemotherapies [44][45][46]. Moreover, the inhibition of autophagy is reported to sensitize CSCs to several anticancer treatment [47]. Unfortunately, the relevance of autophagy inhibition in cancer treatment remains controversial because of the limited availability of chemical modulators. ATG4s are the only cysteine proteases among ATG genes proposed as an attractive candidate to efficiently achieve autophagy inhibition in cancer so far [48]. As an example, ATG4B has been recently proposed has a novel target for leukemic stem cells, supporting the idea that ATG4s proteases are good target for CSCs eradication [49].

Remarkably, we were able to show a correlation between ATM and ATG4C expression in all breast cancer subtypes except for the basal-like one (Table 1) underscoring a clinical impact of our findings. This evidence suggests also that the elucidation of the molecular mechanism whereby ATM regulates ATG4C and autophagy in breast cancer can be relevant as it may pave the way for the development of new biomarkers for diagnostic and/or prognostic evaluation and for the design of novel therapeutic strategies.

## MATERIALS AND METHODS

### DNA constructs, antibodies and reagents

The shATM construct, generously provided by Y Lerenthal and Y Shiloh, had the following sequence: shATM construct (#1 position 912 5′-GACTTTGGCTGTCAACTTTCG-3′) and shATM (#3 position 8538 5′ GGA GCG CAC CAT CTT CTT C 3′) shRNA and control shGFP 5′- GGAGCGC ACCATCTTCTTC-3′ [50]. The ATG4C siRNA was from Signaling Silence ®,Cell Signaling. ATG4C expression construct pCMV3-C-HA was from Sino Biological Inc (HG16060-CY). pEGFP-C3(Clontech).The following antibodies and reagents were used: anti-ATM (2C1; Santa Cruz Biotechnology), anti-tubulin (Sigma, St. Louis, MO, USA), Mouse anti-c-ErbB-2 protein monoclonal antibodies, clone 3B5 (Ab-3, Oncogene Science,Uniondale, NY), anti-ATG4C (Cell Signaling, Beverly, MA, USA), anti-Hsp90 (F8, Santa Cruz Biotechnology), anti-LC3 (Cell Signaling, Beverly, MA, USA), anti-p62 (Santa Cruz Biotechnology), anti-Actin (Sigma, St. Louis, MO, USA), anti-GAPDH (Chemicon). Cloroquine (Sigma, St. Louis, MO, USA), anti-ATG4A (Biorbyt United Kingdom), anti-ATG7 (Cell Signaling, Beverly, MA, USA), Anti-ATG5 (Cell Signaling, Beverly, MA, USA), anti-Cathepsin D (Scripps laboratories, San Diego CA).

### Cell cultures, transfection and infection

Human breast cancer cell lines MCF-7 and MCF-7HER2, described in Stagni et al 2015 [6], were cultured in RPMI-1640 containing 2 mM L-glutamine and supplemented with 10% *H*yClone* Fetal Bovine Serum (Invitrogen) at 37°C in a CO_2_ incubator (5%) . ATM was silenced in MCF7 and MCF7-HER2 cells by lentivirus mediated expression of short-hairpin RNA using lentivirus produced in HEK 293T cells by cotransfecting pSIN18.cPPT.RNAi. p.EGFP.WPRE lentiviral vector with targeting sequences together with respective plasmids encoding for gag-pol and VSV-G proteins. Viral supernatant was collected 48 h post-transfection, filtered through a 0.45 μm pore size filter and added to the cells (MCF7 and MCF7-HER2 breast cancer cell lines) in the presence of 2 μg/ml polybrene [8]. Cells were transiently transfected using Lipofectamine 3000 (Life Technologies) essentially following the manufacturer's instruction.

### Mammosphere culture

Single cell suspensions of breast cancer cell lines, MCF7 and MCF7-HER2, were grown in ultralow attachment 6-well plates (Corning) at a density of 4000 cell/mL in mammosphere medium [Dulbecco's modified Eagle's medium/F- 12, containing 5 ug/mL insulin (Sigma), B27 (Invitrogen), 20 ng/ml epidermal growth factor (GIBCO), 10 ng/ml basic fibroblast growth factor (GIBCO) and 0,4% Bovine Serum Albumine (Sigma)] as described in Dontu *et al*. 2003 [20]. After 7 days, the diameter of mammospheres were measured in phase contrast picures using the I.A.S. software (Delta Sistemi, Rome, Italy). Numbers of the mammospheres (diameter>50 μM) were counted and the efficiency of mammosphere formation was evalueted (%SFE= number of mammospheres / number of plated cells * 100). Mammospheres pellet was collected by gentle centrifugation (900 rpm, 5 min) to further analysis or dissociated into single cell by trypsin addiction (5 min 37°C) and mechanical pipetting. Single cells were plated at a density of 600 cell/well in mammosphere medium supplemented with 5% Fetal Bovine Serum (*H*yClone, Invitrogen) to test the clonogenic activity. After 10 days the cells were stained with 3-[4,5-Dimethylthiazol-2-yl]-2,5-Diphenyltetrazolium Bromide (MTT Vitality Stain, promega) 5 mg/ml for 16 hours and the colony number enumerated. Mammopheres diameter was measured usig I.A.S. software (Delta Sistemi, Rome, Italy).

### ALDH activity assay

To measure and isolate cells with high ALDH activity, the Adelfluor assay was performed according to manufacturer's (Stemcell Technologies, Durham, NC) guidelines. Dissociated single cells were suspended in Aldefluor assay buffer containing the ALDH substrate, Bodipyaminoacetaldehyde (BAAA) at 1,5 μM and incubated for 40 minutes at 37 °C. To distinguish between ALDH-positive and -negative cells, a fraction of cells was incubated under identical condition in the presence of a 10-fold molar excess of the ALDH inhibitor, diethylamino benzaldehyde (DEAB). This results in a significant decrease in the fluorescence intensity of ALDH-positive cells and was used to compensate the flow cytometer (FACScanto, Becton Dickinson).

### RNA extraction and analysis

Total RNA was extracted from breast cancer cell lines MCF7 and MCF7-HER2 and derived mammospheres with TRIzol (Invitrogen) according to the manufacturer's instructions. RNA quantitation was performed via quantitative real-time PCR (RT-PCR). The total RNA was reverse-transcribed with SuperScript III reverse transcriptase (Invitrogen), and amplified by using the Power SYBR Green PCR Master Mix (Applied Biosystems) and the 7900HT Fast Real- Time PCR System (Applied Biosystems). Primers were designed from the Roche Universal Probe Library and were as follows:

**Table:** 

Gene name	Primer sequence 5′ - 3′
hsa-ANL For	TCCCTAGAAGAAGCTGAAGCAG
hsa-ANL Rev	TTCAATTCATCAATCAAAAGTGTTC
hsa-ATG4A For	ACAGATGAGCTGGTATGGATCCTT
hsa-ATG4A Rev	AGACGAGCACTTATATCAGACAACA
hsa-ATG4B For	ATTGGTGCCAGCAAGTCAA
hsa-ATG4B Rev	GCAGGCCAGATGTGAAGG
hsa-ATG4C For	GCATAAAGGATTTCCCTCTTGA
hsa-ATG4C Rev	GCTGGGATCCATTTTTCG
hsa-ATG4D For	ACGTTTCTCAGGACTGCACA
hsa-ATG4D Rev	ACAGACTTCCACTCGGCTGT
hsa-ATM For	TTGTTGTCCCTACTATGGAAATTAAG
hsa-ATM Rev	AGCGAAATTCTGCTTTAAATGAC
hsa-ECT2 For	AGTAAAAGATCTTCCCTTTGAACCT
hsa-ECT2 Rev	CTCGGGCATCCATTTGAA
hsa-HER2 For	TCCTGTGTGGACCTGGATGAC
hsa-HER2 Rev	CCAAAGACCACCCCCAAGA
hsa-HMOX1 For	GGCAGAGGGTGATAGAAGAGG
hsa-HMOX1 Rev	AGCTCCTGCAACTCCTCAAA
hsa-HSPA6 For	CCGCCTATTTCAATGACTCG
hsa-HSPA6 Rev	ATTGATGATCCGCAACACG
hsa-NEDD9 For	GAGCTGGATGGATGACTACGA
hsa-NEDD9 Rev	AGCTCTTTCTGTTGCCTCTCA
hsa-SPRR2A For	TCAACAGCAGCAGTGCAAG
hsa-SPRR2A Rev	CTGTGGACACTTTGGTGGTG
hsa-PLK4 For	GAAAACCAAAAAGGCTGTGGT
hsa-PLK4 Rev	TGAGATGCATACTCCTTTACAAGC
hsa-RAD51 For	AATCCAAATGTAATGCTTTGGTG
hsa-RAD51 Rev	AGGACTGAGATTCTGATTTGACG
hsa-SOX2 For	GGCAGCTACAGCATGATGCAGGAGC
hsa-SOX2 Rev	CTGGTCATGGAGTTGTACTGCAGG
hsa-TBP For	TGCCCGAAACGCCGAATATAATC
hsa-TBP Rev	TGGTTCGTGGCTCTCTTATCCTC
hsa-NANOG For	CAGCTGTGTGTACTCAATGATAGATT
hsa-NANOG Rev	ACACCATTGCTATTCTTCGGCCAGTTG
hsa-OCT4 For	GACAACAATGAAAATCTTCAGGAG
hsa-OCT4 Rev	CTGGCGCCGGTTACAGAACCA

Relative changes in gene expression were quantified by applying the comparative threshold method, also called 2^−ΔΔCt^ method, after determining the Ct values for the reference gene (TBP, the endogenous control) and the target genes in each sample set. All reactions were performed in triplicate. Numerical data were expressed as mean±s.d.

### Protein extraction and western blot analysis

Cells pellet were incubated in RIPA buffer (50 mM Tris-Hcl pH 7.4, 1% NP-40, 0,5% Sodium Deoxycholate, 0,1% SDS, 150mM NaCl, 2mM EDTA, 1mM phenylmethylsulfonyl fluoride, 25mM NaF, 1mM orthovanadate, 40 mM beta-glycerophosphate, 10 mg/ml TPCK, 5 mg/ml TLCK) 30′ on ice and centrifugated at 12000 rpm 10′ a 4°C. For immunoblotting, 20 μg of protein extract were separated by SDS–polyacrylamide gel electrophoresis, blotted onto nitrocellulose membrane and detected with specific antibodies. All immunoblots were revealed by enhanced chemiluminescence (Amersham). Quantification of western blot were determinated using ImageJ software. All statistical analyses were performed with GraphPad Prism 5 software (GraphPad Software, San Diego, CA, USA),using the Student's t-test, P<0.05 being considered significant.

### Immunofluorescence

Cells were washed in PBS and fixed with 4%paraformaldehyde in PBS for 30 min. After permeabilization with 0.4% TritonX-100 in PBS for 5 min or with Digitonin 50μg/ml (in the case of Cathepsin D staining) for 5 min, cells were blocked in 3% normal goat serum in PBS and incubated overnight at 4°C with primary antibodies. We used the antibodies directed against LC3. Cells were then washed in blockingbuffer and incubated for 1 h with labelled anti-rabbit (FITC or Cy3, Jackson ImmunoResearch, West Grove, PA, USA) secondary antibody. Nuclei were stained with 1 mg/ml DAPI and examined under a Zeiss LSM 700100 oil-immersion objective (Zeiss, Oberkoechen, Germany). We used ‘ZEN2009 Light edition’ software for image analysis. All measurements in this workwere performed by a blind approach. All analyses were performed innonsaturated single z-confocal planes.

### Microarray hybridization and data analysis

The microarray assay was conducted using a biological triplicate and two different target sequence for ATM to avoid off targets effects. In particular three independent experiments were performed as follow:

1 Mammospheres MCF7 and MCF7-HER2 infected by lentivirus mediated expression of shGFP as shRNA control or shATM#1.

2 Mammospheres MCF7 and MCF7-HER2 infected by lentivirus mediated expression of pSin18 empty vector as shRNA control or shATM#1.

3 Mammospheres MCF7 and MCF7-HER2 infected by lentivirus mediated expression of shGFP as shRNA control; or shATM#3 .

Mammospheres pellet were collected after 7 days of culture. Total RNA was extracted with TRIzol (Invitrogen) according to the manufacturer's instructions. RNA quality was assessed with an Agilent Bioanalyzer RNA 6000 Nano kit; 200 ng of RNA was labeled with Low Input Quick Amp Labeling Kit, One-Color (Agilent Technologies), purified and hybridized overnight onto, the Agilent 8×60K whole human genome oligonucleotide microarrays (Grid ID 039494) according to the manufacturer's instructions for one-color protocol. The Agilent DNA microarray scanner (model G2505C) was used for slide acquisition and spot analysis was performed with Feature Extraction software ver 10.7 (Agilent Technologies).

### Data analysis

Data filtering and analysis were performed using Microsoft Excel and R-Bionconductor. All the features with the flag gIsWellAboveBG=0 (too close to background) were filtered out and excluded from the following analysis. Filtered data were normalized by aligning samples to the 75^th^ percentile. Differentially expressed genes were selected by a combination of fold change and 1-tail T-test thresholds (p-value<0.05; fold-change ratio >1.5 or <1/1.5 in linear scale and FCR is >0.58 in logaritmic scale). The analysis of over- and under- represented functional annotations was performed using the DAVID web tool [51]. Hierarchical sample clustering and heatmaps were obtained by TM4 MultiexperimentViewer [52]. Updated microarray probe annotations were downloaded from the official Agilent website (https://earray.chem.agilent.com). Array data are deposited in private status on Gene Expression Omnibus databasehttps://www.ncbi.nlm.nih.gov/geo, record GSE81360.

### Comparative analysis with cancer genome atlas

From the TCGA data portal (http://cancergenome.nih.gov), we downloaded all breast cancer expression data files (in the aggregated gene symbol format, for 17814 genes in total), including all metadata [30]. The whole dataset includes 511 primary tumours samples (metastatic and normal tissue samples were excluded), grouped into subtypes according to the Pam50 signature [29] : basal-like n=95, HER2-enriched n=58, luminal A n=231, luminal B, n=127). Within this dataset, we computed the Pearson correlation index, using R-Bioconductor, between ATG4C and ATM genes and all expression TCGA data divided into tumours subtypes.

### Statistical analysis

All statistical analyses and graphs were performed with GraphPad Prism 5 software (GraphPad Software, San Diego, CA, USA). All data are represented as mean±SD. Mean values and standard deviation were generated from at least three biological replicates.For comparision between two groups the Student's t-test was used, P<0.05 being considered significant.

## SUPPLEMENTARY MATERIALS FIGURES AND TABLES


