# Supplementary material for: ATM kinase sustains breast cancer stem-like cells by promoting ATG4C expression and autophagy
Source: Oncotarget. 2017 Feb 20;8(13):21692–709. doi: 10.18632/oncotarget.15537 (PMC5400616; doi:10.18632/oncotarget.15537)
Supplement: Supplementary file 1 [file oncotarget-08-21692-s001.pdf]

ATM kinase sustains breast cancer stem-like cells by promoting ATG4C expression and autophagy

SUPPLEMENTARY MATERIALS

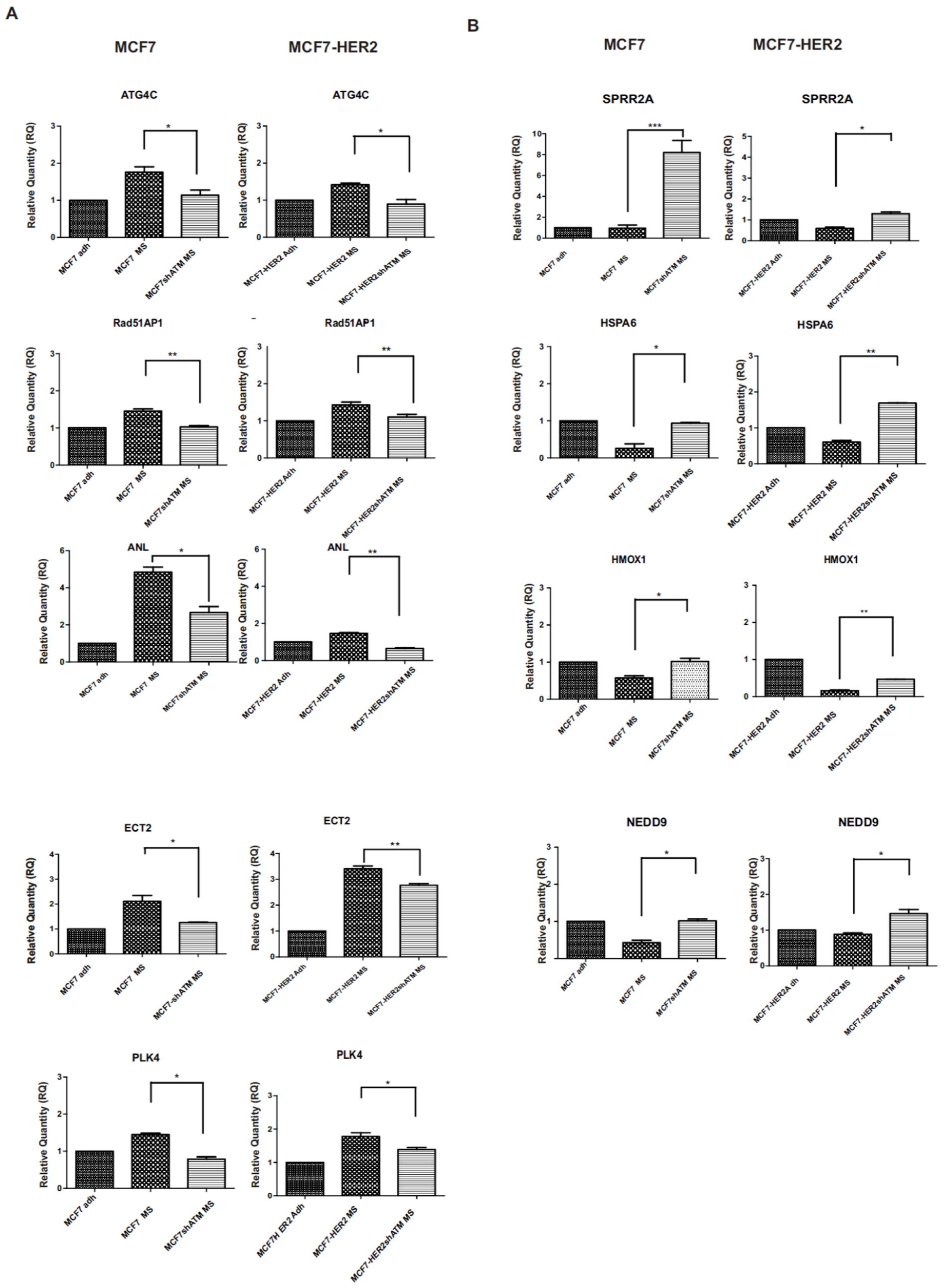

Supplementary Figure 1: The expression of the selected genes in Mammospheres (MS) and Adherent (Adh) cells was investigated using by quantitative Real-Time PCR (RQ, relative quantity). Relatives quantities (RQ) were calculated to TBP and are relative to adherent parental cells (Adh). Results are expressed as the mean±s.d. for at least three independent experiments and analyzed using Student's t-test (*P<0.05, **P<0.01, ***P<0.001). In **A.** genes that are downregulated in mammospheres silenced for ATM compared to control mammospheres and in **B.** genes that are upregulated in mammospheres silenced for ATM compared to control mammospheres.

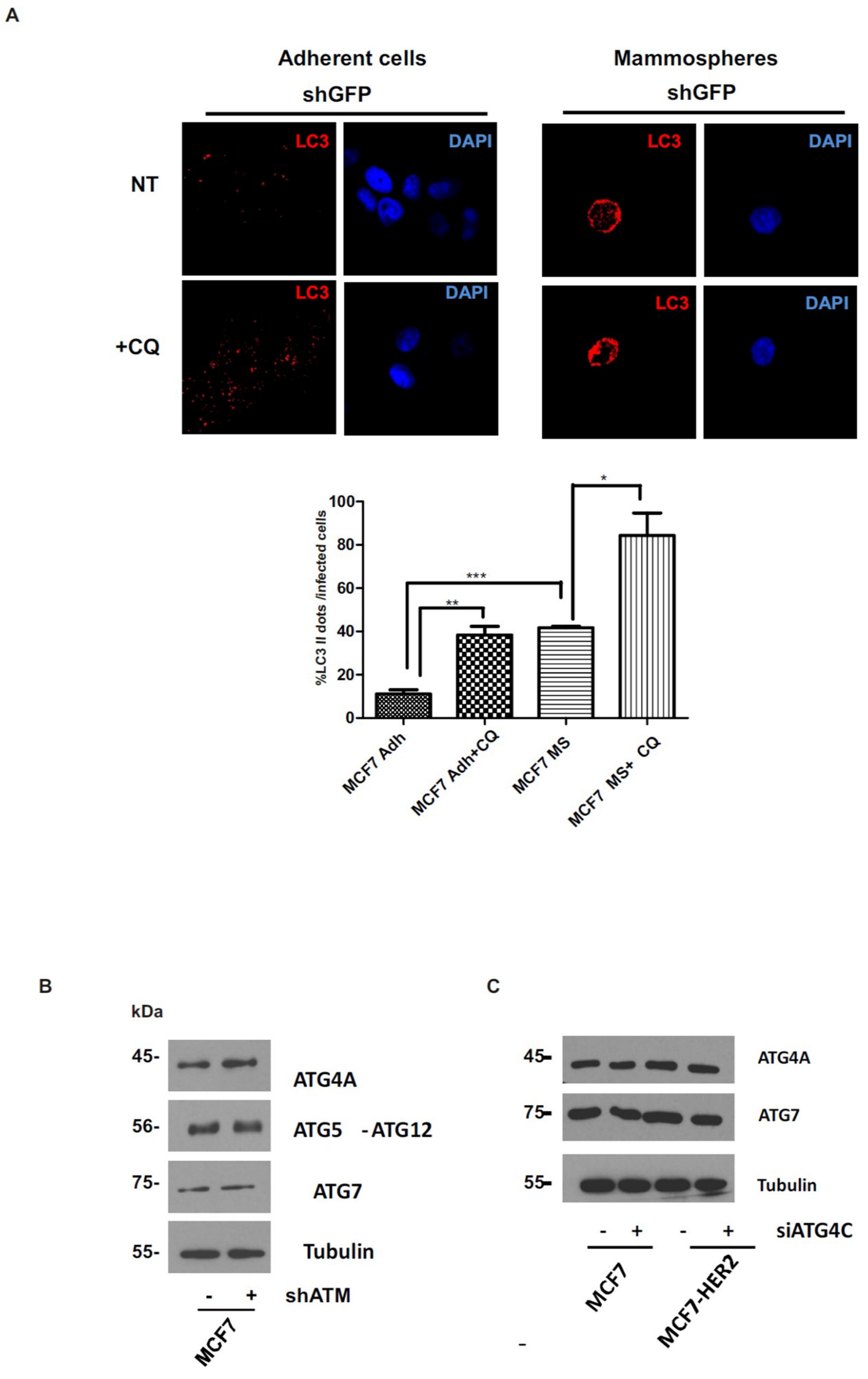

Supplementary Figure 2: A. Representative image of the formation of autophagosome assayed by immunofluorescence for endogenous LC3 protein in MCF7 cells seeded from dissociated mammospheres, using confocal microscopy. Cells were treated or not with CQ (20 μM, 30 min) for analysing autophagic flux. The graph show the accumulation of LC3 dots per infected cells. Results are expressed as the mean±s.d. for at least three independent experiments and analyzed using Student's t-test (*P<0.05, **P<0.01, ***P<0.001). **B.** Representative Western Blot analysis of ATG4A, ATG7and ATG5-ATG12 complex protein levels in mammospheres (MS) with or without shATM. Tubulin was used as loading control**.** **C.** Representative Western Blot analysis of ATG4A and ATG7 protein levels in mammospheres (MS) with or without siATG4C. Tubulin was used as loading control.

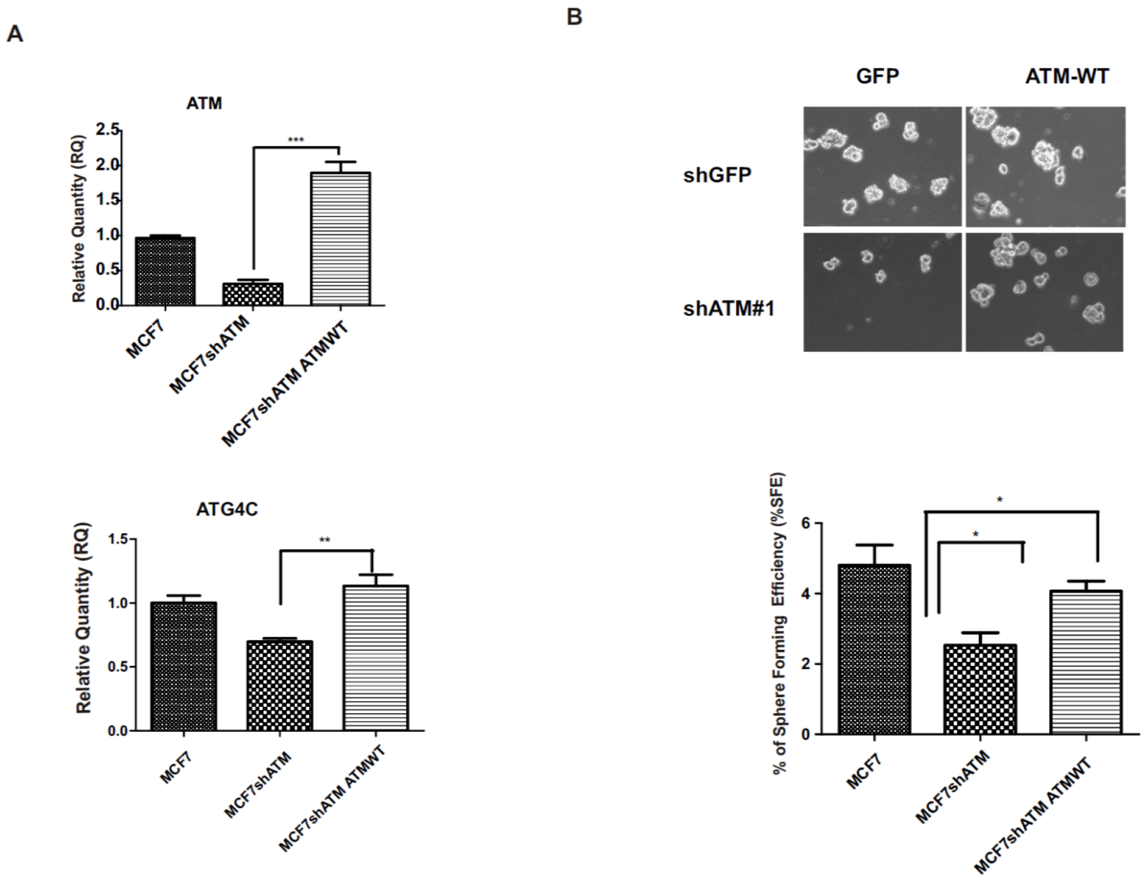

Supplementary Figure 3: MCF7 and MCF7-HER2 breast cancer cell lines transfected with construct overexpressing human pCDNA3-hATM-FLAG (ATMWT) (kindly provided by M. Kastan) and control GFP overexpressing construct, using Lipofectamine ® 3000 Reagent (Life Technologies) **A.** The expression of ATM and ATG4C mRNA levels in Mammospheres (MS), was investigated by quantitative Real-time PCR. Relatives quantities (RQ) were calculated to TBP and are relative to control MCF7 mammospheres. Results are expressed as the mean±s.d. for at least three independent experiments and analysed using Student's t-test (*P<0.05, **P<0.01, ***P<0.001). **B.** Single cells were plated in ultralow attachment plates as described in Materials and methods section, so that cells with stem cell properties were allowed to grow as non-adherent spheroids(mammospheres). Images of the mammospheres were captured on day 7. Representative phase-contrast images of mammospheres are shown (upper). Bars denote 50 µM. Numbers of the mammospheres (diameter>50 µM) were counted, and the %SFE was calculated based on the numbers of cells that were initially seeded (Bottom). Mean±s.d. for three independent experiments performed with both targeting sequences for ATM (shATM#1 and shATM#3) and analysed using Student's t-test (*P<0.05, **P<0.01, ***P<0.001).

Supplementary Table 1: The raw data for each experiment showing the percentage of ALDELFLUOR positive cells

%ALDH positive cells	MCF7	MCF7shATM	MCF7-HER2	MCF7-HER2shATM
Experiment 1	4,81	1,83	14,6	9,94
Experiment 2	5,43	3,51	15,2	10,05
Experiment 3	5,12	2,67	24	9,2
Experiment 4	-	-	23	13,08

Supplementary Table 2: Log2 fold change in array data and Gene Ontology of selected genes in mammospheres shATM versus shCTR. Selected genes validated by *qRT-PCR* shown in bold font

Gene-Symbol	Expression fold change (log2) array		Gene ontology
	MCF7	MCF7-HER2	
ATM	-0.98	-1.40	Cell cycle phase,DNA Repair, regulation of Apoptosis,Mitosis
**ATG4C**	**-1.04**	**-0.72**	**Regulation of Apoptosis, nutrient deprivation**
**Rad51AP1**	**-0.79**	**-1.07**	**DNA repair**
**ANLN**	**-0.8**	**-0.69**	**M phase, Mitosis, Cell cycle phase**
RFC3	-0.63	-0.86	DNA repair, Cell cycle phase
DEPDC1B	-0.67	-0.68	Regulation of protein kinase cascade
TOP2A	-0.64	-0.63	DNA repair, regulation of apoptosis
**ECT2**	**-0.6**	**-0.66**	**M phase, Mitosis, Cell cycle phase**
**PLK4**	**-0.62**	**-0.59**	**M phase, Mitosis, Cell cycle phase**
CCDC15	-0.59	-0.64	M phase, Mitosis, Cell cycle phase
**SPRR2A**	**1.31**	**1.9**	**Epithelial cell differentiation**
**HSPA6**	**1.82**	**1.25**	**Regulation of Apoptosis, stress response**
IL29	1.68	1.68	Regulation of protein kinase cascade, inflammation
SPRR1A	2.44	1.17	Epithelial cell differentiation
ATF3	1.6	1.14	Cell cycle phase, stress response
**HMOX1**	**1.44**	**0.91**	**Regulation of Apoptosis,inflammation**
NEDD9	1.33	0.75	M phase, cell migration, Mitosis
MUC5AC	0.89	1.1	Epithelial cell differentiation
HSPB8	0.64	1.22	Regulation of Apoptosis, stress response
DUSP13	0.79	1	Regulation of protein kinase cascade, M phase
